# Comparative Study between the Combination of Dexamethasone and Bupivacaine for Third Molar Surgery Postoperative Pain: A Triple-Blind, Randomized Clinical Trial

**DOI:** 10.3390/jcm10215081

**Published:** 2021-10-29

**Authors:** Francisco Javier Quesada-Bravo, Ana Rocío García-Carricondo, Fernando Espín-Gálvez, Carmen Fernández-Sánchez, Damaso Fernández-Ginés, María del Mar Requena-Mullor, Raquel Alarcón-Rodríguez

**Affiliations:** 1 Maxillofacial Surgeons of Department of Oral and Maxillofacial Surgery, Torrecardenas University Hospital, 04009 Almeria, Spain; javierquesadabravo@gmail.com (F.J.Q.-B.); argcarricondo@hotmail.es (A.R.G.-C.); maxilofeg@gmail.com (F.E.-G.); 2 Department of Clinical Pharmacology, Torrecardenas University Complex, 04009 Almeria, Spain; carmen.fernandez.s.sspa@juntadeandalucia.es (C.F.-S.); fdamaso-fernandez@gmail.com (D.F.-G.); 3 Department of Nursing, Physiotherapy and Medicine, University of Almería, 04120 Almería, Spain; mrm047@ual.es

**Keywords:** third molar surgery, bupivacaine, dexamethasone, submucosa, postoperative pain

## Abstract

*Objectives:* To compare the possible benefits of the combination of dexamethasone–bupivacaine with articaine–epinephrine as an anaesthetic block after third molar surgery. *Materials and Methods:* Triple-blind, randomized, controlled, parallel, phase 3 clinical trial. Two groups: experimental (93 patients) with standard anaesthetic block: 40/0.005 mg/mL articaine–epinephrine and submucosal reinforcement with 0.8 mg dexamethasone–5% bupivacaine; and control group (91 patients) with standard block: 40/0.005 mg/mL articaine–epinephrine. The surgery consisted of the extraction of the impacted mandibular third molar by performing a procedure following the same repeatable scheme. The visual analogue scale (VAS) was used to analyse postoperative pain. *Results:* Groups were homogeneous, without significant differences related to epidemiological variables. Postoperative pain among the first, second, and seventh postoperative days was statistically significantly lower in the experimental group compared to the control group (*p* < 0.001). Drug consumption was lower in the experimental group throughout the study period (*p* < 0.04). *Conclusion:* Bupivacaine is an alternative to articaine in oral surgery, being more effective in reducing postoperative pain by reducing patients’ scores on the VAS as well as their consumption of analgesic drugs after surgery.

## 1. Introduction

Dentally impacted teeth are very prevalent, with a high incidence of complications: inflammation, infections, cysts, tumours, and so forth. These require specialized treatment, with surgical extraction generally indicated in most cases [1].

In our society, of the dental impactions that are symptomatic or have a pathology in Caucasians, the most common by far affect the lower wisdom teeth, representing nearly 60–70% of the surgical dental extractions and dentoalveolar surgeries performed [1].

The aetiology of abnormalities in tooth development is related to the coexistence of inherent genetic components and specific environmental conditions under which odontogenesis occurs. The main reasons for dental impaction include insufficient space in the dental arch and/or unfavourable angulation, unfavourable position of the tooth germ, atypical development of the impacted tooth, mechanical obstacles, bone scars, gingival fibromatosis, reincorporation of homonymous deciduous teeth, and ankylosis dental. Tooth impaction can have systemic causes such as endocrine disorders, vitamin D deficiency, and hereditary factors. The latest studies published by Trybek et al. showed the indisputable molecular basis of dental defects, which, although complex and polygenic, seems to have a direct relationship with protein products related to genes including MSX1 [2].

In more than 90% of cases, surgical extraction of the lower wisdom tooth is performer or completed under local anaesthesia as outpatient or major outpatient surgery, either in an operating room or a dental office [1].

Postoperative complications from third molar surgery after the surgical trauma and release of local mediators are well known, with the most common being noninfectious complications such as pain, inflammation, and locoregional oedema, as well as limited mouth-opening ability with consequent functional restriction that limits speech and mastication. This postoperative course has a significant influence on the life of the patient [3]. Infectious complications in immunocompetent patients are observed in less than 4% of cases and are mainly related to surgical wound infections. Among these complications, the most common appear in the form of abscess or osteitis, with systemic complications (for example, infective endocarditis or an infection of the implanted artificial joint) being rarer [4]. In the case of third molar surgery, the pain is greatest at first, around 3–5 h postextraction, as a result of the increase in pain mediators as the effect of the local anaesthesia used for the surgery wears off [5]. For this reason, studies have been carried out to demonstrate that it is possible to achieve a longer painless postoperative period using long-acting local anaesthetics [6,7]. Subsequently, the duration of the general symptoms varies from these first few hours to 10–15 days later. As a rule, this pain is controlled with oral analgesics, but long-lasting anaesthetics may also be used [8]. With the mouth, the most commonly used anaesthetic is articaine because of its ideal characteristics: fast onset, potency, and intermediate duration [9,10].

Although studies have shown that corticoids are generally effective [11,12], corticoids have the disadvantage of possible systemic effects that can occur, which derive from the suppression of the adrenocorticotropic hormone (ACTH). Dexamethasone has been shown to be the most effective corticoid, with the ACTH suppression dose being 1.5 mg/day and the cortisone suppression dose 35 mg/day [13].

Given the above, the objective of the present trial was to compare the efficacy of the combination of dexamethasone–bupivacaine on postoperative third molar surgery pain versus articaine–epinephrine as the sole anaesthetic block.

## 2. Materials and Methods

### 2.1. Study Design

**Figure 1:**
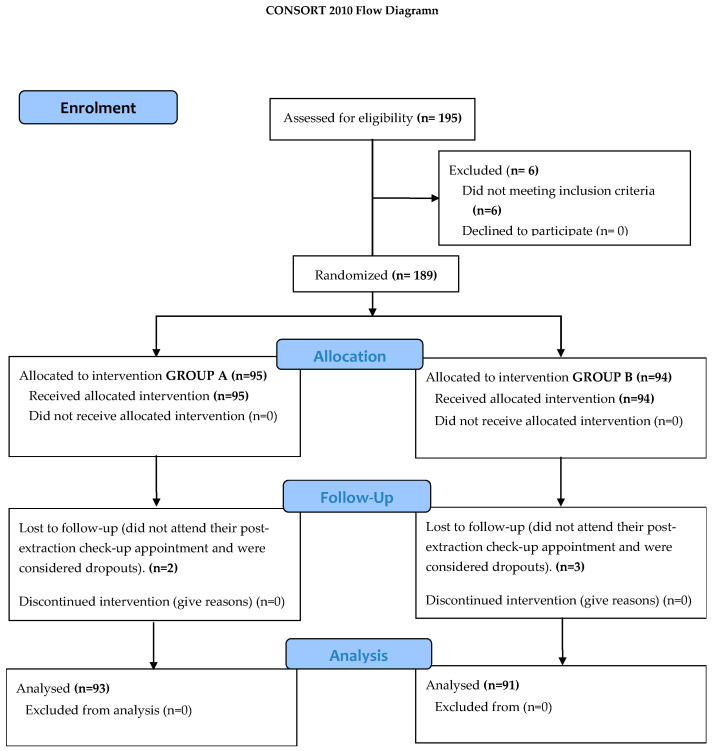
Study design (CONSORT diagram).

This was a triple-blind, randomized, controlled, parallel, phase 3, single-centre clinical trial carried out at the Torrecárdenas Hospital Complex in Almeria, Spain, from May 2015 to October 2016 by the Oral and Maxillofacial Surgery Service. The study was conducted according to Consolidated Standards of Reporting Trials (CONSORT) guidelines (Figure 1) (CONSORT, 2010).

This trial was registered under EudraCT code 2014-000996-01 and was approved by the Torrecárdenas Hospital Research Ethics Committee for the Spanish Agency for Medications and Healthcare Products (protocol code number FJQ-BUP-2014-01, version 2; dated 28 November 2014), and for the Andalusian Biomedical Research Ethics Coordinating Committee (Code CCEIBA 0100/14).

### 2.2. Sampling Size

The sample size was calculated. Since there were no previous data on the mean intensity of pain with anaesthetic blockade with articaine–epinephrine (40/0.005 mg/mL) and dexamethasone (0.8 mg)–bupivacaine (0.5%), a random sample of patients from the experimental group (*n* = 34) and the control group (*n* = 35) was taken to calculate the sample size. The anaesthetic technique and a pain scale were applied to all subjects. The sample size required was determined by comparing the mean between both groups using the Ene 3.0 computer programme. Considering an average pain score for the control group of 3 and for the experimental group of 2.5, with a standard group deviation of 1.5, a potency of 0.8, and a significance level of 0.05, a sample size of *n* = 91 was obtained for both groups, the experimental and the control group.

The inclusion criteria were: 1. male and female adults between 18 and 45 years old (the age range in which tooth eruption or the different pathologies resulting from a lack of or incomplete eruption usually occur) with an indication for surgical dental extraction according to the criteria established in the Andalusian Health Service Dental Impaction Guide; 2. patients with previous dental symptoms (for example, acute pericoronitis, pain, or caries of the second or third molar due to malposition or impaction resulting in the retention of food) [14]. Steed et al. concluded that treatment of the third molar is indicated when symptoms or illnesses can be clearly attributed to this tooth [15]. Patients with incompletely erupted or fully or partially submucous third molars, with a Koerner index for the degree of difficulty of extraction with values of 5–10, obtained by the sum of the Pell and Gregory and Winter classifications [16], were considered as extractions of moderate/severe difficulty. A single extraction was completed per patient, under local anaesthetic, with prior consent to participate in the study.

The exclusion criteria refused patients younger than 18 years old or older than 45 years old; patients with disabilities, allergies, and/or intolerances to the medications used in the study; multiple tooth extraction required during the same surgery; need for general anaesthesia; patients undergoing anticoagulant, oncological, or bisphosphonate treatment; patients with any systemic pathology that could affect the effect; patients with wisdom teeth and associated odontogenic cysts or active infection; patients who had received locoregional radiation therapy or prior surgery on the lower retromolar trigone; pregnant women; and anyone who did not wish to participate in the study or did not authorize their participation.

Recruitment was conducted in the dental office, with previous confirmation of the inclusion criteria via anamnesis and orthopantomography. After patients signed the informed consent form to participate in the trial, randomization was conducted with a 1:1 ratio for the two treatment groups. The EPIDAT 3.1 (Epidemiological Analysis from Tabulated Data) computer system for balanced groups was used to randomize the sample and assign a code to each patient. Participants were distributed into two groups. The first group, the experimental group or group A, received the studied anaesthetic block: 40/0.005 mg/mL articaine–epinephrine and 0.8 mg dexamethasone–0.5% bupivacaine; meanwhile, the second group, the control group or group B, received the standard anaesthetic block: 40/0.005 mg/mL articaine–epinephrine.

Once each patient’s code was obtained, a pharmacist prepared physically identical capsules containing one of the two different drug mixtures and delivered them to the surgical team immediately after surgery. Neither the surgeon, nor the patient, nor the researcher knew the contents of the envelope. The pharmacist recorded every patient code and the treatment assigned in a database.

### 2.3. Surgical Protocol and Measurement Instruments

**Figure 2:**
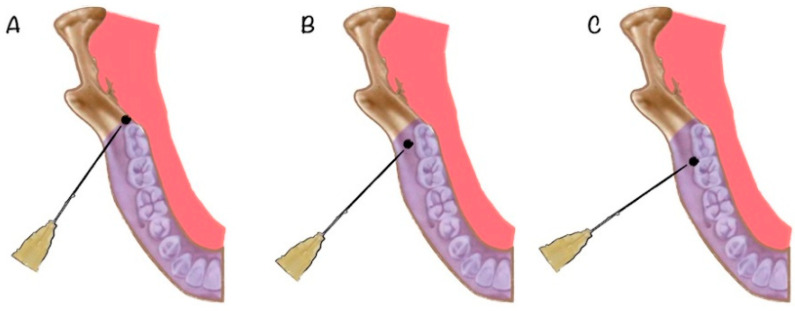
Illustration of the technique used. Presurgical periodontal submucosal infiltration of the blinded cartridge mixture ((**A**) submucosal injection at the distal projection of the third molar; (**B**) submucosal injection in vestibular aspect of the lower third molar; (**C**) submucosal injection in mesial aspect of the lower third molar).

The procedure was carried out by the same surgical team, always using local anaesthetic and electively. The local anaesthesia techniques for extracting lower wisdom teeth have been clearly described in the literature and consist of trunk anaesthesia of the inferior alveolar nerve and homolateral lingual nerve, as well as infiltration anaesthesia of the periodontal mucosa [1] using syringes, needles, and anaesthesia cartridges designed for that purpose. The most used local anaesthetics were derived from amides, such as mepivacaine, articaine, or lidocaine, in association (or not) with vasoconstrictive substances, such as epinephrine or noradrenaline. The prepared anaesthetics came in individual 1.8 cm^3^ cartridges and were injected using the syringes and needles described below. For this study, the anaesthetic block of the oral cavity before surgery consisted of trunk anaesthetic of the inferior alveolar nerve (near the mandibular foramen) and lingual nerve (as the needle was withdrawn) and periodontal anaesthesia of the buccal nerve around the second and third molars using the common anaesthetic of 1.8 mL of 40/0.005 mg/mL articaine with epinephrine (Ultracain^®^) with a stainless steel carpule syringe fitted with a long 30G 0.4 × 38 mm needle (Octoplus^®^). The procedure was the same for all the patients. The periodontal and buccal nerve anaesthesia was then reinforced before dental extraction with three punctures: a submucosal distal puncture of the lower second molar delivering 0.6 cm^3^, a submucosal vestibular puncture of the lower wisdom tooth delivering 0.6 cm^3^, and a submucosal distal puncture of the lower wisdom tooth delivering 0.6 cm^3^ (Figure 2). The experimental group received the study mixture of 0.8 mg dexamethasone diluted in 1.62 mL bupivacaine at 0.5%, while the control group had the same procedure but received 40/0.005 mg/mL articaine with epinephrine.

The anaesthetic technique was followed by extraction of the wisdom tooth with a bayonet flap incision, mucoperiosteal flap, and osteotomy with odontosection, using a round bur with a straight handpiece with continuous irrigation with saline solution [17,18]. Once the process was completed, the surgical site was washed and sutured using absorbable material (Vicryl Rapide− 3/0 Polyglycolic acid, Ethicon^®^) with a single point on the vertical line of the incision and a double point on the posterior line. Finally, an intraoral compression cap was placed with sterile gauze.

After surgical extraction, the patients were given a postoperative instruction sheet with local measures (soft diet, apply local cold, wound tamponade, good oral hygiene) and medical treatment: anti-inflammatory drugs, analgesics, and antibiotics to reduce postsurgical recovery times. In this case, patients were prescribed antibiotic treatment with 1 g amoxicillin or 300 mg clindamycin in case of allergy, along with 600 mg paracetamol or ibuprofen every eight hours for seven days, and a follow-up appointment was scheduled.

All information was confirmed and recorded in the patients’ charts. The information in the case report form (CRF) was registered in a database for subsequent analysis. The patients were seen one week after surgery in the Oral and Maxillofacial Unit’s own outpatient clinic for a follow-up and for data collection.

At the postoperative evaluation appointment, the following information was collected from each patient: the visual analogue scale (VAS) to record postoperative pain, in which each patient rated their pain on a scale from 0 to 10 at 24 h, 48 h, and 7 days after surgery (in a form that on a form that could be filled in at any time); and a tracking sheet to record the consumption of analgesics and anti-inflammatories, on which each patient listed daily the number of analgesics and/or anti-inflammatory pills taken after surgery. Patients who did not meet the inclusion criteria after being selected and those who did not return or deliver the necessary documentation during the postoperative check-up were excluded from the study.

### 2.4. Data Analysis

A database was prepared with the variables collected in the study, and statistical analysis was performed using the IBM SPSS (Statistical Package from the Social Sciences) 23.0 program [19]. First, a descriptive analysis of continuous variables expressed as means and standard deviations was carried out. For categorical variables, frequencies and percentages were explored. Subsequently, the Kolmogorov–Smirnov test was performed to determine whether the continuous variables followed a normal distribution or not. Variables between groups were examined using the independent Mann–Whitney U test for continuous variables and the χ^2^ test of independence for categorical data. Finally, the mean values of the related variables (24 h, 48 h and 7 days) within each group were compared using the Wilcoxon test. A value of *p* < 0.05 was considered statistically significant.

## 3. Results

A total of 195 patients were selected between May 2015 and October 2016, of whom six were withdrawn prior to randomization for not meeting the inclusion criteria. Of the 189 randomized patients, 5 did not attend their postextraction check-up appointment and were considered dropouts, leaving a total of 184 participants. These were divided into two groups according to the mixture applied as periodontal consolidation anaesthetic prior to extracting the lower wisdom tooth. Group A, the experimental group (*n* = 93), received the studied anaesthetic combination, 0.8 mg dexamethasone diluted in 1.62 mL bupivacaine at 0.5%, while Group B, the control group (*n* = 91), received the standard anaesthetic of 40/0.005 mg/mL articaine with epinephrine.

The average patient age was 27.51 (±5.57 SD), with a minimum of 19 and maximum of 40 years old, and 57.6% of patients were female. No significant differences were observed between the two groups in relation to sociodemographic characteristics. Regarding the location of the treated wisdom tooth, in 51.1% of the cases, it was located on the right side of the mouth, and in 48.9% of the cases, on the left side. In group A (experimental), 55.3% were located on the right side, and in group B (control), 44.7%; there was no statistically significant difference between the groups (*p* = 0.18) (Table 1).

Regarding the characteristics of the third molar, the most common alterations in the eruptive process were the retained molar (group A: 52.7%, group B: 53.8%) and the impacted molar (group A: 47.3%, group B: 46.2%). According to the Pell and Gregory classification, the most frequent class and position was IIB, both in group A (26.9%) and in group B (26.4%), followed by IIIA (group A: 22.6%, group B: 20.9%). For Winter’s classification, the vertical position was predominant, with 51.6% in group A and 49.5% in group B; the horizontal position was the second most frequent, with 41.9% and 41.8% in groups A and B, respectively. These results were not statistically significant (Table 1).

The Koerner index of extraction difficulty was elevated in 49.5% of the third molars in group A and in 50.5% of those in group B, with no statistically significant differences observed (*p* = 0.88) (Table 1).

The surgical time calculated from the start of surgery with anaesthetic infiltration and the end with compression packing was 10.64 (±1.90 SD) minutes for group A and 10.88 (±2.33 SD) minutes for group B, these differences not being statistically significant (Table 1).

Regarding the degree of postoperative pain, it was observed that the intensity of pain was lower in patients in group A (experimental) compared to those in group B (control) when comparing the mean values obtained at 24 h, 48 h, and 7 days after surgery following extraction. These results were statistically significant (*p* < 0.001). (Table 2).

When comparing the degree of pain according to gender, in the experimental group, the mean values obtained at 24 h and 48 h were slightly higher in women compared to men, and at 7 days, they were higher in men than in women. However, these differences were not statistically significant. In the control group, women showed higher mean pain intensity values than men at 24 h, 48 h, and 7 days after surgery, with statistically significant differences (Table 2).

Table 3 shows a comparison of the mean scores of pain intensity at 24 h, 48 h, and 7 days between patients with anaesthetic blockade of 40/0.005 mg/mL articaine–epinephrine and dexamethasone 0.8 mg–bupivacaine 0.5%. (experimental group) and patients with anaesthetic blockade of 40/0.005 mg/mL articaine–epinephrine (control group). A decrease in pain intensity was observed 7 days after extraction within each group; these results were statistically significant both in the experimental group and in the control group (*p* < 0.001).

When comparing the mean number of anti-inflammatory/analgesic tablets consumed per patient during the 7 days of follow-up, in group A, the mean intake of paracetamol of 1 g was 0.90 (±2.60 SD) tablets / week. The consumption of paracetamol was higher in group B at 2.01 (±5.38 SD) tablets / week. These differences were statistically significant (Table 4).

The Spearman correlation coefficient of age and duration of the intervention to remove the third molar with the degree of pain at 24, 48 h, and 1 week, was not statistically significant in either of the groups.

## 4. Discussion

Impacted third molars are a common disorder that often requires the extraction of the tooth. Third molar surgery involves local tissue damage, characterized by hyperaemia, vasodilatation, increased capillary permeability with the accumulation of interstitial fluid, and granulocyte and monocyte migration [20]. This is due to higher osmotic pressure in the capillaries (Starling’s law), which produces the most common postoperative consequences, such as pain, trismus, and swelling, all of which are related to the local inflammatory reaction that occurs, in which cyclooxygenase (COX) and prostaglandins play a crucial role. For this reason, there can be serious postoperative discomfort even when the surgical extraction necessitates a simple technique [5]. The literature has described how these complications are influenced by various factors, including the difficulty of the procedure, the age and gender of the patient, and the surgeon’s experience [21]. Regardless, good surgical technique and gentle tissue handling can minimize postoperative inflammation.

Postoperative pain after the extraction of an impacted third molar is often used to evaluate the quality of an analgesic because of the constancy and intensity of this pain [22]. Damage to the soft tissues and the maxillary and mandibular alveolar bones releases inflammatory and analgesic mediators in peripheral sites. Predictably, 3 to 4 h after the operation, at least 95% of patients suffer from moderate-to-severe pain, which can be used to distinguish active medication from placebos in clinical trials and can also detect differences between modern, highly effective analgesics [23]. The degree of extraction difficulty was high in 49.5% of the third molars in group A and 50.5% of those in group B. The malposition of vertical impaction in position IIB was the most frequent in both groups, as in the study from Bhargava et al. [24]; in our case, this accounted for 26.9% and 26.4% of cases in groups A and B, respectively.

As the field of pharmacology has evolved, many medications have been used to alleviate postsurgical pains, with both peripheral and central analgesia demonstrating utility. A combination of analgesics can have a “sparing effect” such that a lower dose of the drugs can relieve pain with fewer side effects. As many nociception pathways exist in the human body, a combination of medications is recommended by the World Health Organization (WHO) [25]. NSAIDs (nonsteroidal anti-inflammatory drugs) have been shown to be effective in managing postoperative dental pain. A commonly used medication in this category is ibuprofen [26]. The efficacy of ibuprofen in treating postoperative dental pain has been evaluated in several clinical trials [8,21,26]. It suppresses both PGE2 (prostaglandins E2) and TXB2 (thromboxanes 2), having a dual inhibitory effect on COX-1 and COX-2 cyclooxygenase isoforms [22].

Because of the frequent development of noninfectious complications such as gastrointestinal bleeding, decreased kidney function, etc., therapies and techniques to reduce the severity of the postsurgical clinic and prevent pharmacological side effects have been created. Among them are the technique of applying platelet-rich fibrin to the postextraction socket, which has been shown to reduce symptoms as described in the study by Trybek et al. [27], and the use of Kinesio taping, presented by Jaroń et al. [4], which has been shown effective in reducing postoperative oedema, pain, and trismus after impacted mandibular wisdom teeth surgery.

Corticoids must be administered before tissue damage in order to reach the proper tissue level in the immediate postoperative period [28]. One of the lesser-studied types of administration of corticosteroids is via submucosal administration [12]. The few studies published on this route have shown that the submucosal administration of corticosteroids such as dexamethasone controls postoperative oedema and pain. According to Majid et al. [29], the submucosal administration of 4 mg or 8 mg of dexamethasone 1 h before surgery improved oedema and pain when compared with nontreated groups, with no differences observed between the two dosing regimens. This was congruent within the study by Grossi et al., in which 4 mg submucosally administered dexamethasone had an effect on postoperative oedema, but increasing the dose did not produce any change or benefit. The authors noted that this technique has a limited, nonsignificant effect concerning postoperative pain [30]. In our study, the administered dose of intraoperative submucosal dexamethasone was 0.8 mg, and differences in terms of pain were found between the experimental and control groups.

Corticosteroids are used primarily after surgical procedures to suppress inflammatory tissue mediators; this suppression reduces the transudation of liquids and decreases subsequent oedema. Although it appears that this reduction of oedema is accompanied by some reduction in postoperative pain, it is thought is that steroids alone do not have a clinically significant analgesic effect [12,31]. The use of steroids may conversely increase a patient’s susceptibility to pain by suppressing endorphin levels. Dionne et al. showed that 4 mg dexamethasone administered orally at 12 h preoperatively and an additional 4 mg administered intravenously 1 h before third molar surgery did not sufficiently suppress the release of PGE2 to produce an analgesic effect after local anaesthesia offset. Their findings did not support the use of glucocorticoids as an analgesic following third molar surgery [31]. However, other studies have shown that dexamethasone seemed to decrease pain after surgery [32], suggesting that steroids may be related to a reduction in the number of analgesic tablets taken after surgical extractions. Our study supported this hypothesis, since statistically significant differences were found in terms of drug use between the experimental and control groups during the seven-day follow-up.

Gersema et al. [33] and Milles et al. [34] could not demonstrate that the administration of steroids significantly reduced pain, although a non-statistically significant decrease was observed in the perception of pain by patients between the control group and the experimental group, as occurred in the studies by Pederson et al. [35] and Graziani et al. [13]. Conversely, in their study comparing the use of intramuscular and submucosal dexamethasone against a placebo group, Majid et at. [29], did demonstrate that both treatment groups showed significant reductions in swelling and pain when compared to the control group at every interval. These findings were congruent with our study, in which patients treated with submucosal dexamethasone perceived less pain at all study intervals. Grossi et al. [30] and Markiewicz et al. [36] elaborated by observing that the patients in the experimental group who took corticosteroids reported less early postoperative pain (days 1–3) but not less late pain (days 4–7) compared to the control groups. In our study, the pain was lower in the experimental group on the days evaluated during the follow-up process.

Other recent studies, such as the works by Sancho-Purchades et al. [9], Fernández et al. [37], and Olmedo et al. [8], have more firmly verified this evidence, showing lower pain levels in patients treated with bupivacaine when compared with those treated with lidocaine and mepivacaine. Indeed, in a study comparing articaine at 4% with bupivacaine at 0.5%, Thakare et al. [38] indicated that the former provided better pain control.

Statistically, our study is different from those by Pellicer-Chover et al. [39] and Brković et al. [5], which found no clear statistically significant differences in postoperative pain after the extraction of wisdom teeth using different types of anaesthetics. In our study, the patients who received only the articaine block reported initial pain immediately after the anaesthetic wore off, while those with bupivacaine reported it later. There were statistically significant differences in postoperative pain between both groups.

The average age of the patients in our study with pathology associated with the third molar was 27.51 and, as reported by Venta et al. [40], the number of symptom-free wisdom teeth decreases with age. In our study, the ages ranged between twenty and thirty years old, the time of wisdom tooth eruption when the most pericoronitis occurs. Pericoronitis is a pathology more common with the third molar (64%) in subjects below the age of 30 and is responsible for 66% of surgical extractions [41]. Generally speaking, young females with a high education level have been associated with a higher absence of illness in the third molar [42].

With regard to distribution by gender, 57.6% of the patients were women and 42.4% men, values that differ from those in the study by Grossi et al. [30], which worked with 46% women and 54% men. According to some studies, gender influences the perception of pain. The same literature speculates that women have been described as feeling pain more intensely than men, so it appears that women are five times more likely to have complications as pain [43]. The dose of endogenous oestrogen changes during the menstrual cycle, which influences the fibrinolytic process and anti-inflammatory response during days 23 to 28 of the menstrual cycle [44]. Our study did not observe any differences in the degree of pain in men and women between the experimental groups, as was observed by M.J. García et al. [44].

As Grossi et al. [30] stated, an increase in the dose of corticosteroids did not provide any benefit regarding postoperative complications. The results from this study provide a basis for the submucosal administration of low doses of corticosteroids such as dexamethasone to reduce postoperative pain and oedema. Presumably, the injection of a low dose of dexamethasone in the surgical site achieves a greater effective concentration of the medication in the site of the lesion, without loss to other compartments due to extravasation or the onset of elimination [29]. Moreover, when the third molar is surgically removed with local anaesthesia, use of the submucous route is convenient for both the surgeon and the patient, since the efficacy of the oral administration route depends on the patient’s compliance and requires correct adherence to subsequent treatment, which is not always achieved, to maintain optimal blood levels during the postoperative period.

As for strengths, a clinical trial designed with the triple blindness was conducted and the performance of the procedure by a single team. Moreover, no significant differences were found in the different variables, as homogenous groups were involved. Randomization minimized selection bias and increased the possibility of prospectively obtaining a wider patient characteristic range. One of the weaknesses of the study could be that bupivacaine has an intermediate onset speed and latency time [32], while its high liposolubility reduces its efficacy in infiltration techniques, because a large amount is retained in the soft tissues and only part of it reaches the bone. However, the amount absorbed produces a longer-lasting anaesthetic effect, relieving local pain.

## 5. Conclusions

In summary, this study provides a basis for the use of the mixture of anaesthetic and corticoids via submucous infiltration as a simple, painless, noninvasive (because the submucosal infiltration is done when the standard anaesthetic block has already been effected), and profitable treatment for any cases of pain [24]. The preoperative injection has the advantage of concentrating the medication near the surgical area with less systemic absorption, less loss through distribution to other compartments, and slower elimination, making it possible to provide a local deposit of anaesthesia in the tissue as a means to reduce pain. Furthermore, for both the surgeon and the patient, it is convenient that submucous administration is more effective, since oral consumption depends on the patient’s adherence to treatment, which may not be constant, and since, if it is to work correctly, oral medication requires a constant dose in the blood, which repeated administration does not always achieve.

This study presents a long-term, safe, and effective postoperative analgesic, a finding that can be used for surgical procedures in which the pain can be controlled with a complementary submucosal or perilesional injection of bupivacaine and dexamethasone before surgery.

## Figures and Tables

**Table 1:** Comparison of sociodemographic data and characteristics of the third molar between the two groups, A (experimental) and B (control).

Characteristic		Group A (Experimental) (*n* = 93)	Group B (Control) (*n* = 91)	*p* Value
Age, in years ^a^		26.77 ± 5.77	28.26 ± 5.30	0.07 *
Sex	Male	39 (41.9%)	39 (42.9%)	0.89 **
	Female	54 (58.1%)	52 (57.1%)	
Laterality	Right	52 (55.9%)	42 (46.2%)	0.18 **
	Left	41 (44.1%)	49 (53.8%)	
Pell and Gregory	IIA	17 (18.3%)	19 (20.9%)	0.98 **
	IIIA	21 (22.6%)	19 (20.9%)	
	IB	14 (15.1%)	12 (13.2%)	
	IIB	25 (26.9%)	24 (26.4%)	
	IIIB	16 (17.2%)	17 (18.7%)	
Winter	Vertical	48 (51.6%)	45 (49.5%)	0.83 **
	Horizontal	39 (41.9%)	38 (41.8%)	
	Mesioangular	6 (6.5%)	8 (8.8%)	
Koerner	Moderate	47 (50.5%)	45 (49.5%)	0.88 **
	High	46 (49.5%)	46 (50.5%)	
Eruptive alteration	Impacted	44 (47.3%)	42 (46.2%)	0.87 **
	Retained	49 (52.7%)	49 (53.8%)	
Surgical time (minutes) ^a^		10.64 ± 1.90	10.88 ± 2.33	0.77 *

*p* value obtained with * Mann–Whitney U test or ** χ^2^ test. ^a^ Results are expressed as Mean ± SD.

**Table 2:** Comparison of the mean scores for the degree of pain from 24 h, 48 h and 1 week.

Type of Anaesthetic Technique		Pain 24 h	*p* Value	Pain 48 h	*p* Value	Pain 1 Week	*p* Value
Group A (Experimental)		1.65 ± 0.86	<0.001 *	1.26 ± 0.99	<0.001 *	0.69 ± 0.83	<0.001 *
Group B (Control)		3.70 ± 2.25		3.08 ± 2.6		1.97 ± 1.72	
Group A (Experimental)	Male	1.62 ± 0.84	0.78 *	1.18 ± 0.99	0.52 *	0.74 ± 0.78	0.58 *
	Female	1.67 ± 0.89		1.31 ± 1.0		0.65 ± 0.87	
Group B (Control)	Male	3.08 ± 1.69	0.01 *	2.18 ± 1.27	<0.001 *	1.26 ± 1.06	<0.001 *
	Female	4.17 ± 2.51		3.75 ± 2.28		2.50 ± 1.92	

*p* value obtained with * Mann–Whitney U test. Results are expressed as Mean ± SD.

**Table 3:** Comparison of the mean pain at 24 h, 48 h, and a week in the experimental group (A) and the control group (B).

Type of Anesthetic Technique		Pain 24 h	Pain 48 h	Pain 1 Week	*p* Value
Group A (Experimental)	Total	1.65± 0.86	1.26 ± 0.99	0.69 ± 0.83	<0.001 *
	Male	1.62± 0.84	1.18 ± 0.99	0.74 ± 0.78	<0.001 *
	Female	1.67± 0.89	1.31 ± 1.0	0.65 ± 0.87	<0.001 *
Group B (Control)	Total	3.70 ± 2.25	3.08 ± 2.06	1.97 ± 1.72	<0.001 *
	Male	3.08 ± 1.69	2.18 ± 1.27	1.26 ± 1.06	<0.001 *
	Female	4.17 ± 2.51	3.75 ± 2.28	2.50 ± 1.92	<0.001 *

*p* value obtained with * Wilcoxon test. Results are expressed as Mean ± SD.

**Table 4:** Comparison of the number of tablets of analgesics depending on the type of anaesthetic technique in two groups, A (experimental) and B (control).

Characteristic	Group A (Experimental) (*n* = 91)	Group B (Control) (*n* = 93)	*p* Value
Ibuprofen (tablets/week)	12.52 ± 6.99	13.69 ± 8.66	0.31 *
Paracetamol 1g (tablets/week)	0.90 ± 2.60	2.01 ± 5.38	0.04 *
Paracetamol 650mg (tablets/week)	0.36 ± 1.32	0.74 ± 3.73	0.36 *

*p* value obtained with * Mann–Whitney U test. Results are expressed as Mean ± SD.
